# Video data of hydraulic fracture propagation in two-dimensionally confined gelatin plates

**DOI:** 10.1016/j.dib.2019.104096

**Published:** 2019-06-15

**Authors:** Soo-Min Ham, Tae-Hyuk Kwon

**Affiliations:** Department of Civil and Environmental Engineering, Korea Advanced Institute of Science and Technology (KAIST), Daejeon, Republic of Korea

**Keywords:** Hydraulic fracture, Fracture visualization, Fracture propagation video

## Abstract

This data article presents 36 raw and unprocessed video files which were obtained during hydraulic fracture experiments in two-dimensionally confined gelatin plates. Initiation and propagation of bi-wing fractures were recorded at 50 frames per second with the resolution of 1920 × 1080 pixels. The gelatin stiffness, the fluid viscosity, and the flow rate were controlled. With these data, the fracture geometry, such as length, width, and propagation velocity were discussed in the research article “Characteristics of steady-state propagation of hydraulic fractures in ductile elastic and two-dimensionally confined plate media” Ham and Kwon et al., 2019.

**Table:** Specifications Table

Subject area	Earth Sciences
More specific subject area	Geotechnical Engineering/Geology
Type of data	Video
How data was acquired	Acquired with a high-speed camera (UI–3360CP–C-HQ, IDS Corp.)
Data format	Raw
Experimental factors	Video files recorded during hydraulic fracture experiments
Experimental features	The fracture propagation in gelatin was recorded by controlling the gelatin stiffness, fracturing fluid viscosity, and injection flow rates.
Data source location	Daejeon, 34141 Republic of Korea
Data accessibility	Data are available with this article, at website [2]http://kwon.kaist.ac.kr/home/archive_,_ and at Mendeley Data [3]: Ham S, Kwon T. Video data of the hydraulic fracturing propagation in two-dimensionally confined gelatin plate, Mendeley Data, v1 https://doi.org/10.17632/73fpxk7vzb.1.
Related research article	Ham S, Kwon T, Characteristics of steady-state propagation of hydraulic fractures in ductile elastic and two-dimensionally confined plate media, Int. J. Rock Mech. Min. 114 (2019) 164–174 [1].

**Table:** 

**Value of the data** • Data in this article can be used with other sets of similar data to investigate general behaviors of the fracture propagation • Data in this article can be used for comparison with data on the effect of other factors • These data will be useful to researchers working on theoretical modeling of hydraulic fracture or fluid-driven fractures

## 1 Data

This dataset comprises 36 raw and unprocessed video files obtained using the high-speed camera (UI–3360CP–C-HQ, IDS Corp., Korea) during hydraulic fracturing (HF) experiments. The movies are formatted in MP4 format with the frame size of 1920 × 1080 pixels. Conditions for each HF experiment are indicated by the filename: S for the gelatin stiffness, Q for the fluid injection rate, and CP for the fluid viscosity. The videos were recorded from the onset of fluid injection. The bi-wing hydraulic fractures were generated and propagated, and then the experiment was terminated when the fluid injection stopped.

The following are the supplementary data related to this article:

1

2

3

4

5

6

7

8

9

10

11

12

13

14

15

16

17

18

19

20

21

22

23

24

25

26

27

28

29

30

31

32

33

34

35

36

## 2 Experimental design, materials and methods

Gelatin samples were prepared in the acrylic mold, of which the size was 200 × 200 × 5 mm (length × width × thickness), by pouring gelatin solutions and then cured at 4 °C for 20 hours. Thereafter, the copper tube was placed at the center of the plate, and was then connected to the transfer vessel. This gelatin sample was placed under a LED lamp for recording using a high-speed camera (UI–3360CP–C-HQ, IDS Corp.). Bi-wing type of two hydraulic fractures were generated by injecting the fracture fluid dyed with blue ink through the penetrated borehole. The time-lapsed images of hydraulic fracture initiation and propagation were acquired using the high-speed camera facing up the gelatin sample recorded at 50 frames per second with the resolution of 1920 × 1080 pixels. A total of 36 cases were tested with four levels of gelatin stiffness, three classes of fracturing fluid viscosity (1, 10, and 100 cp), and four different flow rates (5, 10, 20, and 40 mL/min). The gelatin stiffness was controlled with the water prior to gelatin concentration: 3.84, 7.41, and 12.28, 13.79 wt% for S0, S1, S2, and S3. Each of the HF experiments is labeled using the symbols representing each test condition: S0, S1, S2, and S3 for gelatin stiffness levels, CP1, CP10, and CP100 for the fracturing fluid viscosity, and Q5, Q10, Q20, and Q40 for the injection flow rate. For example, S0Q5CP1 which is VideoS01_S0Q5CP1 file is the result of the experiment under the conditions of S0 stiffness of gelatin, 5 mL/min of the flow rate, and 1 cp of the fluid viscosity. The detailed experiment procedure and analysis were presented in *Ham and Kwon (2019)* [1].
